# Comparison of Core Stabilisation Exercise and Proprioceptive Neuromuscular Facilitation Training on Pain-related and Neuromuscular Response Outcomes for Chronic Low Back Pain: A Randomised Controlled Trial

**DOI:** 10.21315/mjms2019.26.6.8

**Published:** 2019-12-30

**Authors:** Pattanasin Areeudomwong, Vitsarut Buttagat

**Affiliations:** Department of Physical Therapy, School of Integrative Medicine, Mae Fah Luang University, Chiang Rai, Thailand

**Keywords:** exercise, low back pain, muscle activity, disability, training

## Abstract

**Background:**

Existing literature offers little guidance for therapists who provide core stabilisation exercise (CSE) and proprioceptive neuromuscular facilitation (PNF) training to treat chronic low back pain (CLBP). Studies conducting a head-to-head comparison of CSE and PNF training for CLBP are needed.

**Objective:**

To compare the effects of CSE and PNF training on pain-related outcomes and trunk muscle activity in CLBP patients.

**Methods:**

Forty-five CLBP patients, ranging from 18 to 50 years of age, were randomly divided and assigned to either a four-week CSE, four-week PNF training, or control group. Pain-related outcomes, including pain intensity, functional disability and patient satisfaction, as well as superficial and deep trunk muscle activity were assessed before and after the four-week intervention, and at a three-month follow-up.

**Results:**

Compared to the control group, those in the CSE and PNF training groups showed significant improvements in all pain-related outcomes after the four-week intervention and at three-month follow-up (*P* < 0.01). Following the four-week intervention, both CSE and PNF training groups demonstrated significant improvement in deep trunk muscle activity, including the transversus abdominis (TrA) and superficial fibres of lumbar multifidus (LM), compared to the control group (*P* < 0.05).

**Conclusion:**

Four-week CSE and PNF training provided short-term and long-term effects on pain-related outcomes, along with increased deep trunk muscle activity in CLBP patients.

## Introduction

Chronic low back pain (CLBP), defined as individuals who experience pain between the 12th rib and inferior gluteal folds for at least 12 weeks, is a global health problem causing suffering, disability and work absenteeism (1). CLBP prevalence in working-age adults is estimated at 19.60% (2). Considering therapeutic costs and lost productivity, the economic burden of CLBP to society is substantial (3).

While CLBP is a major problem worldwide, there is no consensus as to specific causes and over 90% of those with CLBP are diagnosed with non-specific low back pain (LBP) (4). Several causes, including biopsychosocial factors, affect CLBP; however, trunk muscle weakness, particularly deep trunk muscles, along with poor coordination and trunk proprioception, are associated with persistent LBP (5–7). This could pose a greater risk of instability to the lumbar spine, further spine injury, and, ultimately, decreased physical activity (8). Hence, therapeutic interventions that improve deep trunk muscle function and trunk proprioception may improve pain-related and neuromuscular parameters in CLBP patients.

Several interventions, such as exercises and physical modalities, including therapeutic ultrasound which is frequently used to improve lower back symptoms and function (9), have been proposed as optimal treatments for CLBP, but the most effective interventions for CLBP are still discussed (10–12). Exercise therapy is suggested as an effective approach for CLBP (13). Core stabilisation exercise (CSE) is a popular option in restoring functions of trunk muscles to achieve optimal lumbar stability during daily activities (14–17). CSE includes training aimed at re-educating deep trunk muscle function, and coordination of deep and superficial trunk muscles in static, dynamic, and functional tasks (14).

Proprioceptive neuromuscular facilitation (PNF) training is known to improve proprioceptive function within muscles and tendons of the lumbar region, thereby increasing trunk muscle activity and coordination in response to neuromuscular stimuli (10, 18–19). As the diagonal and spiral patterns of PNF are similar to the topographic arrangement of muscles used in daily activities and sports (15), it is believed that PNF training facilitates the function of several muscles better than unidirectional exercises for CLBP relief (10–12, 18, 19). Thus, improving proprioception via PNF training may benefit lumbar stability.

Numerous researchers have reported the effectiveness of CSE (14–17) and PNF training (10–12, 18–19), however current literature offers little guidance for therapists in determining which interventions to implement for CLBP. To our knowledge, randomised head-to-head comparisons of short- and long-term effects of CSE and PNF training on pain-related and electromyographic response parameters in CLBP patients is scarce. Consequently, this study aims to compare the effects of CSE and PNF training on pain-related outcomes, including pain intensity, functional disability, patient satisfaction, and trunk muscle activity in CLBP patients.

## Methods

### Study Design

Designed as an assessor-blinded and randomised controlled trial, the study was approved by the Ethics Committee for Human Research at Mae Fah Luang University (REH 60139) based on the Declaration of Helsinki.

### Participants

Between October 2018 and March 2019, 45 eligible participants with CLBP seeking treatment from Mae Fah Luang University Hospital in Chiang Rai, Thailand were invited to this study. Eligibility criteria consisted of history of CLBP for over 12 weeks, patient age of 18 to 50 years, and pain intensity assessed by over two numerical rating scale (NRS) scores. All participants were screened for illness history and underwent physical examination by a medical doctor unaware of the intervention. Patients were excluded if they had specific spinal pathology (e.g., sacroiliac joint dysfunction, disc herniation), malignancy, neurological compromise, history of lumbopelvic surgery, pregnancy, or regularly received CSE, PNF, trunk strengthening, or ultrasound therapy. Participants provided informed consent before participation.

### Sample Size Estimation

A formula of repeated-measure analysis of variance (ANOVA) was employed for sample size estimation. Our sample size of 45 was estimated to have a 90% chance of detecting differences between groups of two scores on the 11-point NRS at four weeks of intervention (21). An alpha level of 0.05 and a worst-case loss to follow-up of 10% were calculated.

### Therapeutic Interventions

Using simple randomisation by drawing lots, eligible participants were assigned to one of three 15-member groups: CSE, PNF training or control. Randomisation results were concealed in a sealed and opaque envelope. The research assistant performing randomisation was not involved in recruiting participants or providing interventions. Members of each group participated in three weekly 30 min sessions over four weeks at the Physical Therapy Laboratory. Interventions were performed by a neutral researcher, unaware of the outcome measurement. Participants used treatment diaries to keep track of the interventions.

### CSE group

Under physiotherapist supervision, participants practiced recruitment of deep trunk muscles, particularly transversus abdominis (TrA) and lumbar multifidus (LM) muscles, together with the diaphragm and pelvic floor muscles, reducing superficial trunk muscle activity in order to improve function of deep trunk muscles and control inter-segmental lumbar spine movement during activities (22). Initially, participants were taught how to perform isolated contraction of TrA and LM (independent from superficial trunk muscles) using an abdominal drawing-in manoeuvre (ADIM) in conjunction with contraction of pelvic floor muscles in minimum loading positions, such as prone or sitting positions. Furthermore, co-contraction of TrA and LM muscles was performed after isolated contraction of those muscles was achieved. Participants practiced these exercises with a 10 s hold for 10 repetitions in weeks 1 and 2. A pressure biofeedback device (Chattanooga Australia Pty. Ltd., Brisbane, Queensland) and an electromyography biofeedback (MP 36, BIOPAC system, Goleta, California) were provided to participants to guide performance of each muscle. Exercise difficulty was increased by integrating deep muscle co-contraction with controlling movement of extremities and heavier loading positions, such as bridging, bird-dog position and single knee to chest, with a 10 s contraction hold and 10 repetitions in weeks 3 and 4 (23). A 30 s rest between repetitions and a 60 s rest after each set were provided.

### PNF training group

Participants performed 15 repetitions of each PNF training for three sets, with a 30 s rest between repetitions and a 60 s rest after each set. In week 1, participants practiced rhythmic stabilisation (RS), a 10 s hold of alternating isometric contractions of the trunk flexor and extensor muscles in a sitting position against maximum force provided by a physiotherapist. For week 2, participants performed a combination of isotonics (COI) focused on alternating concentric, eccentric and isometric contractions of the trunk muscles in a sitting position; this included a 5 s resisted concentric contraction of the trunk flexor muscle, followed by a 5 s resisted eccentric contraction of the trunk flexors in the return to trunk neutral position and a 5 s resisted isometric contraction of the trunk muscles in a neutral position. The same method was applied for trunk extensors. For weeks 3 and 4, participants were trained in alternately performing the chop and lift (CL) patterns of the upper extremities with maximum resistance (19).

The physiotherapist in the CSE and PNF groups considered each participant’s ability to perform the exercise prior to prescribing exercise progression.

### Control group

Participants received 5 min to 10 min of therapeutic ultrasound depending on treatment area. A frequency of 1 MHz and continuous mode with intensity between 1.5 and 2.5 W/cm^2^ was applied as treatment for CLBP (24). Additionally, the 20-min general trunk strengthening exercise program, which is routine for CLBP and includes trunk curl-up, diagonal curl, and single-leg extension, was performed in three sets of 10 repetitions, with a 30 s rest between repetitions and 60 s rest between sets (25).

Throughout the four-week intervention, participants were asked to report any side effects of the treatment. While participants were asked to avoid pain killers and other treatment during the study period, anyone who needed pain killers or other treatment during the study was require to note this in a personal logbook.

### Outcome Measures

A blinded assessor unaware of the randomisation assessed all outcome measures. The main outcome measure was pain intensity using the 11-point NRS with scores ranging from 0 (no pain) to 10 (extreme pain). Participants circled the numerical value representing their pain level; for CLBP, a clinically significant change is two scores (26).

Secondary outcomes consisted of functional disability, patient satisfaction and neuromuscular response of trunk muscles. The 24-item Thai version of the Roland-Morris Disability Questionnaire was used to evaluate functional disability of LBP. The total score ranged from 0 (no disability) to 24 (maximum disability) (27). The global perceived effect was assessed on an 11-point scale, ranging from −5 (extremely worse) through 0 (no change) to +5 (completely recovered). Participants were asked to compare current back symptoms with the baseline (28).

Surface electromyography (MP 36, BIOPAC Systems, Goleta, CA, USA) was used to measure activations of rectus abdominis (RA), TrA, iliocostalis lumborum pars thoracis (ICLT) and superficial fibres of LM muscles. Skin was prepared to achieve 5 kΩ of impedance before bilateral attachment of eight pairs of surface electrodes over the noted muscles. Electrode placement was in accordance with previous literature (23, 29). Ground electrodes for all pairs were placed on the ipsilateral lower rib cages for RA and ICLT, and iliac crests for TrA and superficial fibers of LM. Electromyographic signals were sampled at 1000 Hz, with gain of 1000 Hz, 30 Hz to 500 Hz bandwidth and 85 dB common-mode rejection ratio (23).

Maximum voluntary isometric trunk flexion was performed in a crook lying position (28), trunk extension was performed using a modified Biering-Sørensen test (23), and a maximal Valsalva and forced expiratory manoeuvre was performed in a supine position (30) for RA, ICLT, superficial fibres of LM and TrA muscles, respectively. Participants performed and held each tested position for 5 s, repeating it three times with a 60 s rest between tests to avoid fatigue (23). The root mean square (RMS) value during the middle 5 s for each test was recorded. Before data collection, the intra-rater reliability test for measurement of muscle activity assessed by the assessor demonstrated high reliability (intraclass correlation coefficient, ICC(3,3) = 0.82–0.90, *P* < 0.01).

Pain intensity, functional disability and patient satisfaction were measured at three periods: baseline assessment, four-week intervention, and three-month follow-up. Activations of RA, TrA, ICLT, and superficial fibres of LM muscles were measured at baseline assessment and four-week intervention only.

### Statistical Analysis

The data was expressed as mean, standard deviation and the 95% confidence interval. The SPSS version 20 (IBM Corporation, Armonk, NY, USA) was used for statistical analysis based on the intention-to-treat approach using the last observation carried forward. The data was found to be normally distributed using the Shapiro-Wilk test. A 2 × 3 (group × time) repeated measures ANOVA was employed to compare between-group differences for pain intensity, functional disability, and patient satisfaction. If significant interaction effects were shown, multiple comparisons would be applied. Within-group comparisons of those outcomes were tested using one-way repeated measures ANOVA.

For trunk muscle activity, one-way ANOVA was used for between-group comparison and paired *t*-test was used for within-group comparison. A *P*-value of less than 0.05 was determined as statistically significant.

## Results

**Figure 1:**
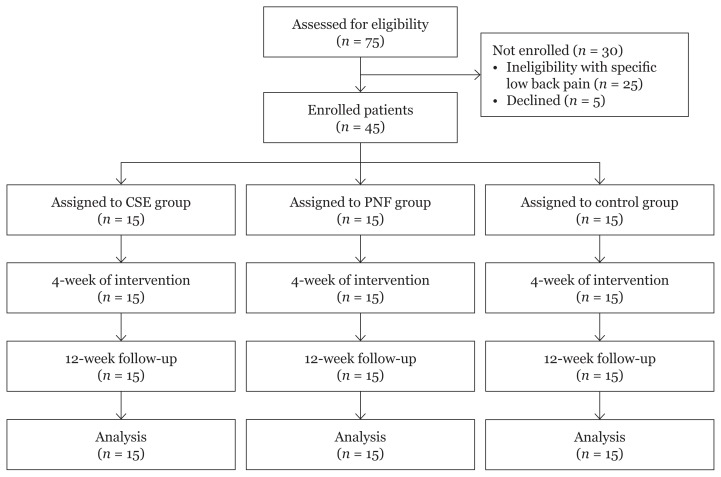
Flow diagram of participants tracking from enrollment to analysis

Forty-five out of 75 CLBP patients met the study’s inclusion criteria, with no attrition rate. Twenty-five patients were excluded from the study due to their specific LBP conditions and five patients were declined (Figure 1). All baseline characteristics were similar between the groups as shown in Table 1. No participants mentioned any side effects during the intervention period.

Significant interaction effects were found on pain intensity (F_(4,56)_ = 7.58; *P* < 0.001), functional disability (F_(4,56)_ = 2.67; *P* = 0.04) and patient satisfaction (F_(4,56)_ = 9.97; *P* < 0.001). Both CSE and PNF training groups showed significantly greater reduction in pain intensity and functional disability than the control group after the completion of the four-week intervention period (*P* < 0.001), and at three-month follow-up period (*P* < 0.05). The CSE and PNF training groups also had better patient satisfaction than the controls (*P* < 0.01) (Table 2). The CSE and PNF training groups showed significant improvements in bilateral activity of TrA and superficial fibres of LM muscles compared with the control group (*P* < 0.05) (Table 3).

**Figure 2:**
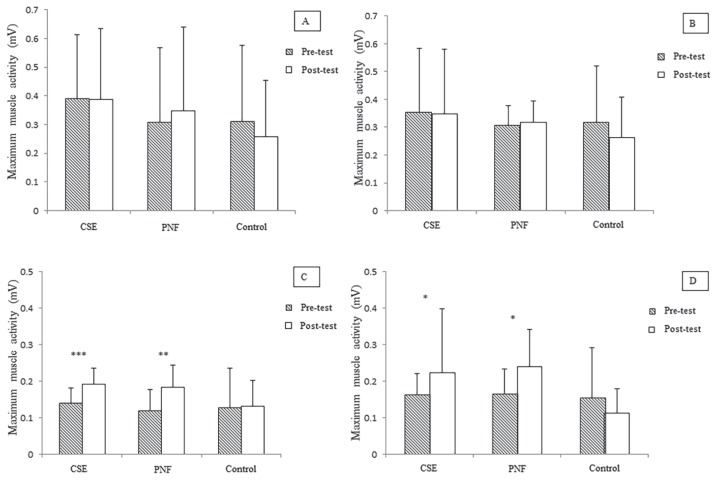
(A) Within-group comparison of maximum muscle activity of right rectus abdominis, (B) left rectus abdominis, (C) right transversus abdominis and (D) left transversus abdominis of CSE, PNF and control groups. *P < 0.05; **P < 0.01; ***P < 0.001 (Paired *t*-test)

**Figure 3:**
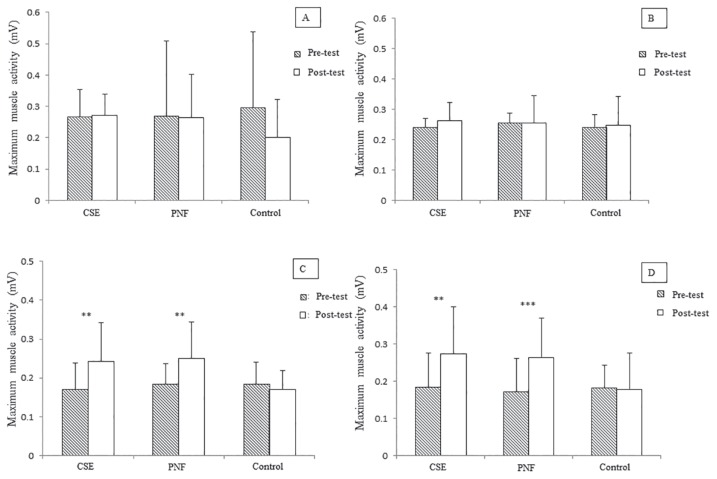
(A) Within-group comparison of maximum muscle activity of right iliocostalis lumborum pars thoracis, (B) left iliocostalis lumborum pars thoracis, (C) right superficial fibres of lumbar multifidus and (D) left superficial fibres of lumbar multifidus of core stabilisation exercise (CSE), proprioceptive neuromuscular facilitation (PNF) and control groups. **P < 0.01; ***P < 0.001 (Paired *t*-test)

For within-group comparisons, compared to baseline, the CSE and PNF training groups demonstrated significantly higher improvement in pain intensity (*P* < 0.001), functional disability (*P* < 0.001), patient satisfaction (*P* < 0.001) (Table 4), and activity of TrA and superficial fibres of LM muscles bilaterally (*P* < 0.05) (Figures 2 and 3) after completion of the intervention. Improvements in pain intensity, functional disability and patient satisfaction were maintained at three-month follow-up (*P* < 0.001). Compared to baseline, the control group had only better patient satisfaction (*P* < 0.001) (Table 4).

## Discussion

This study aimed to compare the effects of CSE and PNF training on pain-related and neuromuscular outcomes in CLBP patients. Overall, compared to the control group, our findings showed that after a four-week intervention, both therapies improved pain intensity, functional disability, and patient satisfaction, as well as activation of TrA and superficial fibres of LM muscles. At a three-month follow-up, it was observed that achieved pain-related outcomes persisted. Greater improvement in those outcomes from baseline to follow-up period were also observed in both CSE and PNF training groups.

While CLBP can have several causes, weakness and poor coordination of trunk muscles, particularly deep trunk muscles (so-called core muscles), and poor trunk proprioception are associated with persistent LBP (5–7). Previous research proposed that improper trunk muscle activation and coordination could cause abnormal excessive intersegmental movement of the lumbar spine (31–33). Furthermore, a disturbance in mechanoreceptors of the trunk and impairment of superior proprioception centers related to motor control may be associated with LBP (7, 34–35). These factors could be causes of lumbar spine instability during activities or sports, resulting in increased pain and decreased functional ability (7, 31–35).

This study found short-term effectiveness of CES on pain intensity, functional ability, and patient satisfaction. The findings are in accordance with the meta-analysis study of Wang et al. (36) which included five potential clinical trials comparing CSE and general trunk exercises. CSE had superior short-term effects for pain reduction and increased functional ability in CLBP patients. The present study also showed significant improvements in activations of TrA and superficial fibers of LM muscles, possibly resulting from CSE. Areeudomwong et al. (23) support our findings that ADIM training of CSE can increase activation of deep abdominal muscles in CLBP patients with clinical lumbar instability. The principle of CSE is to restore the neuromuscular system’s ability to control the spine, thereby preventing injury (14–15). Our study chose ADIM for training as it can improve TrA and LM muscle function and their capacities, such as strength to control intersegmental movements of the lumbar spine (22–23). Moreover, re-education of TrA and LM coordination, as well as superficial trunk muscles, resulting from CSE training could promote controlled mobility of the spine during functional tasks and sports (14, 22–23). Thus, for CLBP patients, the increase in activations of TrA and superficial fibers of LM muscles may result in decreased pain and functional disability, while increasing satisfaction.

This study presented long-term effects of CSE on pain-related outcomes. While we do not know if there was a sustained improvement effect of CSE on deep trunk muscles, it is speculated that better activation of trunk muscles may result in persistent effects for improving pain-related outcomes. The present findings are inconsistent with previous studies finding no greater effectiveness of CSE compared to general trunk exercises (38–39). The difference in findings between the present and preceding studies may be due to methodological variations, including age and severity of LBP symptoms. More homogeneous research trials are required to clarify long-term effects of CSE on pain-related outcomes and trunk muscle activation in CLBP.

Patients in the PNF training group had significant improvements in all pain-related outcomes in short- and long-term follow-ups, concurring with the previous study’s findings regarding pain intensity, functional disability and patient satisfaction in 4 and 12 week follow-ups (19). Increased activations in TrA and superficial fibres of LM muscles or core muscles, seen in this group may be an underlying cause of the improved pain-related parameters of this study. The present study used three techniques of PNF training, including RS, COI and CL, to enhance neuromuscular control of the lumbar spine. Continuous proprioceptive feedback of the trunk and neuromuscular readjustment may be important in controlling trunk movements in daily tasks (40). All techniques of PNF training in the present study were performed in spiral and diagonal patterns, facilitating activation of several muscles over their patterns (18). RS may promote trunk stabilisation due to training trunk muscles isometrically. The dynamic nature of COI, which is performed with concentric, eccentric, and isometric contractions of the trunk muscles, and the CL, which consists of reciprocal contractions of the trunk muscles, may increase trunk muscle coordination and improve trunk proprioception, thus enhancing muscle-controlled mobility in daily life (18, 41). Moreover, the irradiation which is defined as increasing the overflow and the spread of the muscular activity from responding to resistance in specific patterns might receive from resistance-induced temporal or spatial summation (42). Our study speculated that improvement of deep trunk muscle activity may be caused by irradiation from resistance to upper body and limb movements. This is supported by Hwang and Park (43) who examined transversus abdominis/internal oblique muscle activity during ADIM combined with bilateral arm extension. They reported that irradiation due to bilateral arm extension could provide additional benefits for enhancing activity of transversus abdominis/internal oblique muscle.

This study has a few key strengths. As an assessor-blinded randomised controlled trial, measurement and selection bias were minimised (44). All participants fully attended the interventions and follow-up, so attrition was not an issue. To our knowledge, this study is the first head-to-head comparison investigating the effectiveness of CSE and PNF training in CLBP patients, with physical modality plus general trunk exercises as a control.

This study had some limitations. Our population consisted of working-age CLBP patients, whose results may not correlate with other age groups, such as adolescents and older adults, or other LBP conditions, such as spondylolisthesis, spondylosis, and herniated nucleus pulposus. We only investigated effects of CSE and PNF training on pain-related outcomes and electromyographic activity of trunk muscles; future studies should consider other physical or psychological outcomes, such as patient-specific activity or fear avoidance belief. Furthermore, CSE and PNF training were compared to a control group that received therapeutic ultrasound and performed general trunk exercises. Other interventions, such as McKenzie’s exercise, could be considered. Also, long-term effects of CSE and PNF training on trunk muscle activity and irradiation were not investigated; further studies on this should be conducted.

## Conclusion

Four-week CSE and PNF training programmes provide both short- and long-term improvements in pain-related outcomes, including pain intensity, functional disability and patient satisfaction in CLBP patients. Following both interventions, increased deep trunk muscle activity was also demonstrated.

## Figures and Tables

**Table 1:** Baseline characteristics of the participants

Baseline characteristic	PNF group (*n* = 15)	CSE group (*n* = 15)	Control group (*n* = 15)
Age, mean±SD (years)	24.00±8.47	24.08±1.00	24.36±9.97
Female sex, number (%)	12 (80)	11 (73.3)	11 (73.3)
Body mass index; mean±SD (kg/m^2^)	22.88±5.06	21.93±4.32	22.56±3.94
Low back pain duration, mean±SD (month)	6.80±3.97	6.33±3.28	6.36±2.36
Pain intensity, mean±SD (score)	4.13±0.92	4.40±1.40	4.07±1.28
Functional disability, mean±SD (score)	4.53±2.13	4.60±2.17	4.47±2.07
Maximum muscle activity (mV)			
Right rectus abdominis	0.391±0.221	0.309±0.259	0.312±0.265
Left rectus abdominis	0.353±0.230	0.308±0.071	0.317±0.203
Right transversus abdominis	0.140±0.041	0.120±0.057	0.128±0.108
Left transversus abdominis	0.164±0.057	0.165±0.069	0.155±0.137
Right iliocostalis lumborum pars thoracis	0.267±0.087	0.269±0.240	0.295±0.242
Left iliocostalis lumborum pars thoracis	0.243±0.031	0.256±0.033	0.242±0.041
Right iliocostalis lumborum pars lumborum	0.171±0.067	0.184±0.053	0.185±0.056
Left iliocostalis lumborum pars lumborum	0.185±0.090	0.171±0.090	0.182±0.060

PNF = Proprioceptive neuromuscular facilitation, CSE = Core stabilisation exercise

**Table 2:** Comparisons of pain intensity, functional disability and patient satisfaction among PNF (*n* = 15), CSE (*n* = 15) and control (*n* = 15) groups

Outcome	CSE versus PNF	CSE versus control	PNF versus control

	Mean difference a (95% CI)	Mean difference a (95% CI)	Mean difference a (95% CI)
Pain intensity (score)			
Baseline	−0.27 (−1.16 to 0.63)	0.07 (−0.83 to 0.96)	0.33 (−0.56 to 1.23)
4 weeks	0.33 (−0.53 to 1.20)	1.73 (0.87 to 2.60)***	1.40 (0.54 to 2.26)**
3 months	0.27 (−0.52 to 1.06)	2.12 (0.54 to 2.12)**	1.07 (0.28 to 1.86)**
Functional disability (score)			
Baseline	0.07 (−1.63 to 1.50)	−0.07 (−1.63 to 1.50)	−0.13 (−1.70 to 1.43)
4 weeks	0.47 (−1.01 to 1.95)	2.47 (0.99 to 3.95)**	2.00 (0.52 to 3.48)**
3 months	0.07 (−3.33 to 3.50)	4.00 (0.60 to 7.40)*	3.93 (0.53 to 7.33)*
Patient satisfaction (score)			
Baseline	0	0	0
4 weeks	0.40 (−0.36 to 1.16)	0.07 (−0.70 to 0.83)	−0.33 (−1.10 to 0.43)
3 months	0.40 (−0.21 to 1.01)	1.53 (0.92 to 2.14)***	1.13 (0.52 to 1.74)**

Notes:

a The 2 × 3 repeated measures analysis of variance with pairwise comparison

CI = confidence interval, CSE = core stabilisation exercise; PNF = proprioceptive neuromuscular facilitation

* *P* < 0.05;

** *P* < 0.01;

*** *P* < 0.001

**Table 3:** Between-group comparison of maximum muscle activity of the trunk muscles after 4-week of intervention among CSE (*n* = 15), PNF (*n* = 15) and control (*n* = 15) groups

Outcome	CSE versus PNF	CSE versus control	PNF versus control

	Mean difference a (95% CI)	Mean difference a (95% CI)	Mean difference a (95% CI)
Right RA (mV)	0.014 (−0.163 to 0.192)	0.105 (−0.073 to 0.282)	0.090 (−0.087 to 0.268)
Left RA (mV)	0.029 (−0.092 to 0.149)	0.083 (−0.038 to 0.204)	0.054 (−0.067 to 0.175)
Right TrA (mV)	0.009 (−0.035 to 0.054)	0.061 (0.016 to 0.105)**	0.051 (0.007 to 0.096)*
Left TrA (mV)	−0.017 (−0.108 to 0.073)	0.110 (0.019 to 0.201)*	0.127 (0.036 to 0.218)**
Right ICLT (mV)	0.007 (−0.076 to 0.089)	0.071 (−0.073 to 0.282)	0.064 (−0.018 to 0.047)
Left ICLT (mV)	0.007 (−0.076 to 0.089)	0.013 (−0.048 to 0.075)	0.007 (−0.055 to 0.068)
Right LM (mV)	−0.008 (−0.070 to 0.054)	0.073 (0.010 to 0.135)*	0.080 (0.018 to 0.143)*
Left LM (mV)	0.011 (−0.071 to 0.092)	0.098 (0.016 to 0.179)*	0.087 (0.005 to 0.169)*

Notes:

a The one-way analysis of variance (ANOVA);

CI = confidence interval; CSE = core stabilisation exercise; PNF = proprioceptive neuromuscular facilitation; mV = millivolt; RA = rectus abdominis; TrA = transversus abdominis; ICLT = iliocostalis lumborum pars thoracis; LM = superficial fibres of lumbar multifidus;

* *P* < 0.05;

** *P* < 0.01

**Table 4:** Within-group comparisons of pain intensity, functional disability and patient satisfaction of CSE, PNF and control groups at baseline, and 4-week and 3-month follow-ups

Outcome	CSE group (*n* = 15)	Mean difference a (95% CI)	PNF group (*n* = 15)	Mean difference a (95% CI)	Control group (*n* = 15)	Mean difference a (95% CI)

	Pain intensity (score)					
Baseline	4.13±0.92		4.40±1.40		4.07±1.28	
4 weeks	1.73±0.96	2.40±0.27 (1.66 to 3.14)***	2.07±0.88	2.33±0.35 (1.39 to 3.28)***	3.47±1.55	0.60±0.46 (−0.64 to 1.84)
3 months	1.93±1.03	2.20±0.22 (1.60 to 2.81)***	2.20±0.86	2.20±0.30 (1.50 to 3.00)***	3.27±1.28	0.80±0.30 (−0.004 to 1.60)
Functional disability (score)						
Baseline	4.53±2.13		4.60±2.17		4.47±2.07	
4 weeks	1.47±1.60	3.07±0.57 (1.53 to 4.60)***	1.93±1.79	2.27±0.45 (1.43 to 3.90)***	3.93±2.52	0.53±0.26 (−0.16 to 1.23)
3 months	1.80±1.32	2.73±0.44 (1.53 to 3.93)***	1.87±1.56	2.73±0.38 (1.69 to 3.78)***	5.80±7.74	−1.33±2.07 (−6.96 to 4.29)
Patient satisfaction (score)						
Baseline	0		0		0	
4 weeks	3.40±1.06	3.40±0.27 (2.66 to 4.14)***	3.00±0.93	3.00±0.24 (2.35 to 3.65)***	3.33±1.11	3.33±0.29 (2.55 to 4.11)***
3 months	2.73±0.80	2.75±0.21 (2.17 to 3.29)***	2.33±0.90	2.33±0.23 (1.70 to 2.97)***	1.20±0.77	1.20±0.20 (−1.74 to 0.66)

a The one-way repeated measure analysis of variance (ANOVA) with comparison between each time point to the baseline;

CI = confidence interval; CSE = core stabilisation exercise; PNF = proprioceptive neuromuscular facilitation;

* *P* < 0.05;

** *P* < 0.01;

*** *P* < 0.001
